# There are no safe areas for avoiding the perforating arteries along the proximal part of the femur: A word of caution

**DOI:** 10.1002/ca.23398

**Published:** 2019-05-13

**Authors:** Bettina Pretterklieber, Eleonore Pablik, Karl Dorfmeister, Michael L. Pretterklieber

**Affiliations:** ^1^ Division of Anatomy, Center for Anatomy and Cell Biology Medical University of Vienna Wien Austria; ^2^ Section for Medical Statistics, Center for Medical Statistics Informatics and Intelligent Systems, Medical University of Vienna Wien Austria

**Keywords:** anatomy, surgery, orthopedics, traumatology, arteries, femur, vascular system injury, fracture fixation, internal fixators

## Abstract

Knowledge about the variable course of the perforating arteries near the body of the femur is essential during surgical procedures (e.g., percutaneous cerclage wiring, plate osteosynthesis, Ilizarov technique). Our aims were to determine the number of perforating arteries, and to identify safe zones along the body of the femur within which perforating arteries are unlikely to pass toward the back of the thigh. The number of perforating arteries was determined in both legs of 100 formalin‐fixed anatomic specimens of both sexes. The level of passage of perforating arteries near the body of the femur was measured in reference to a line from the anterior superior iliac spine to the medial femoral condyle. In each leg, two to seven perforating arteries were present. In 64% of legs, at least one artery divided into two to four branches before entering the back of the thigh. Thus, the total number of branches passing near the body of the femur varied between two to nine. Perforating arteries passed to the back of the thigh at every level between 14.0 and 36.5 cm from the anterior superior iliac spine (16–39% of the leg length). Within this distance, no safe zones along the body of the femur could be identified. The present study shows the high variability regarding number and course of the perforating arteries. Surgeons can be faced with an artery at every level on the posteromedial aspect of the body of the femur between 14.0 and 36.5 cm distally to the anterior superior iliac spine. Clin. Anat. 33:507–515, 2020. © 2019 Wiley Periodicals, Inc.

## INTRODUCTION

**Figure 1:**
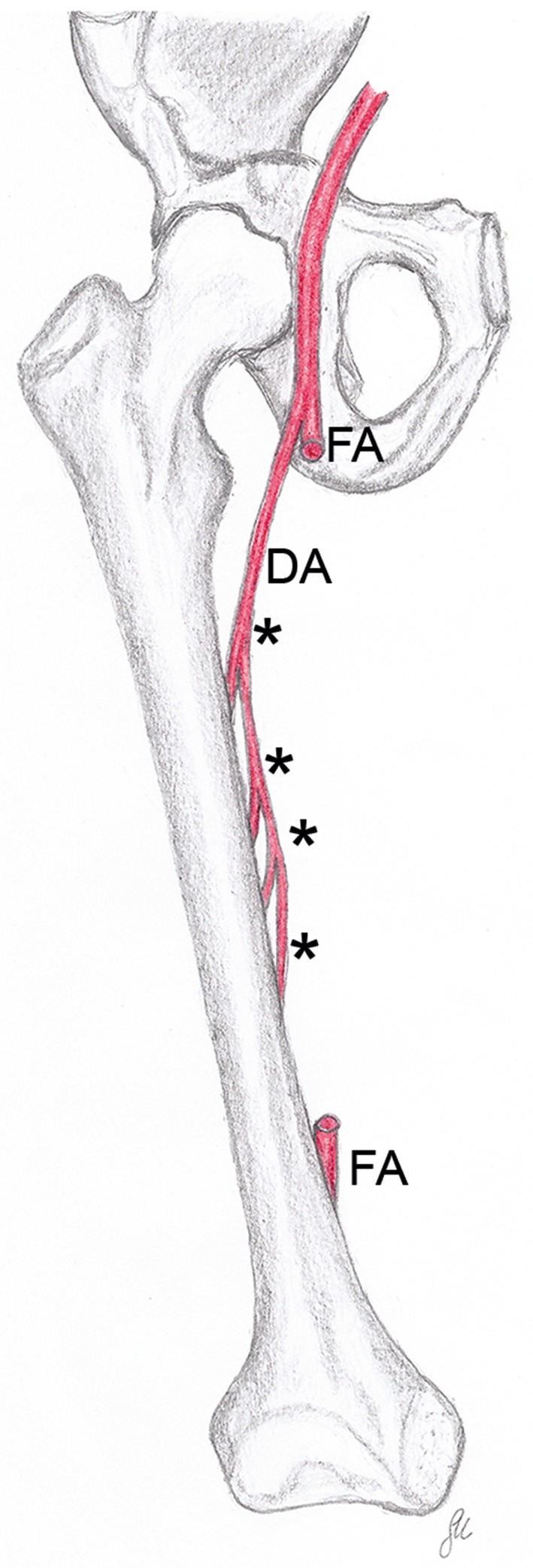
This drawing shows an idealized view of the number, source, and course of the perforating arteries (asterisks) as described in textbooks of anatomy. The femoral artery (FA) has been “resected” in its middle part. DA, deep artery of thigh. [Color figure can be viewed at http://wileyonlinelibrary.com]

Perforating arteries are branches of the deep artery of thigh (Fig. 1). They pass between the tendons of the adductor muscles and the body of the femur to supply the muscles and skin on the back of the thigh, and give rise to one or two nutrient arteries for the body of the femur (Laing, 1953; Williams and Warwick, 1980; Yamamoto et al., 1995). Textbooks of anatomy and surgery commonly describe three to four perforating arteries (Lang and Wachsmuth, 1972; Williams and Warwick, 1980; Netter, 1987; Hoppenfeld et al., 2009). However, numbers, course, and diameters of these arteries have already been reported to vary considerably (Siddharth et al., 1985; Bergmann et al., 1996–2019; Manjappa and Prasanna, 2014). Injury of the perforating arteries during screw insertion, use of retractors, or similar procedures may lead to complications like pseudoaneurysms, lesions in the vessel wall, or vascular occlusion. In the worst case, the patients may lose their leg, or even their life (Neubauer et al., 2016). Therefore, knowledge about the course of the perforating arteries is essential during surgical approaches onto the body of the femur, for example, plate osteosynthesis (Farouk et al., 1997; Farouk et al., 1999; Neubauer et al., 2016), cerclage wiring (Apivatthakakul et al., 2018; Devendra et al., 2018), application of an Ilizarov apparatus (Paley, 1989; Goldstein et al., 2013), or the harvesting of free or pedicled perforator flaps (Yamamoto et al., 1995; Ahmadzadeh et al., 2007).

Only few authors have attempted to identify the exact position of the perforating arteries along the body of the femur. These studies differ methodologically, especially concerning the reference landmarks used (Siddharth et al., 1985; Farouk et al., 1999; Dissanayake et al., 2016). Almost all authors measured the level of origin of the perforating arteries from the deep artery of thigh. The arteries are actually in greater danger at their passage near the body of the femur during cerclage wiring or plate osteosynthesis (Neubauer et al., 2016; Devendra et al., 2018).

The aims of our study were to determine the number of perforating arteries, and to locate safe zones in which perforating arteries unlikely pass close to the body of the femur.

## MATERIALS AND METHODS

### Sample

We examined both legs from 100 formalin‐fixed anatomic specimens (41 males, 59 females) from our student anatomy dissection courses. Their age of death was on average 80.5 years. The deceased individuals had donated their bodies to medical education and research at our department. In addition to the informed consent of the body donors, the study was approved by the ethics committee of our University (EK No. 1102/2018). We included only specimens without any signs of previous surgical interventions, such as arterial prosthesis, stents, or bypasses along the femoral artery (FA) or the deep artery of thigh.

### Dissection

The femoral triangle was dissected carefully to expose the deep artery of thigh and its branches. We removed all veins except the femoral and great saphenous veins. An arterial branch of the deep artery of thigh was classified as a perforating artery if it ran laterally or laterodistally and passed through the tendinous arch of insertion of the adductor muscles to enter the back of the thigh. As the perforating arteries frequently divided into two to four branches before their passage near the body of the femur, we also determined the number of these branches. In addition, we calculated the difference between arteries originating from the deep artery of thigh and branches passing near the body of the femur. The terminal branch of the deep artery of thigh was also considered as a perforating artery if it entered the posterior femoral region between the tendinous arch of the adductor magnus and the body of the femur. As the terminal part of the deep artery of thigh regularly descends between the adductor longus and magnus muscles, we had to detach the proximal part of insertion of the adductor longus muscle to determine the exact position of the arteries' passage.

### Measurements

**Figure 2:**
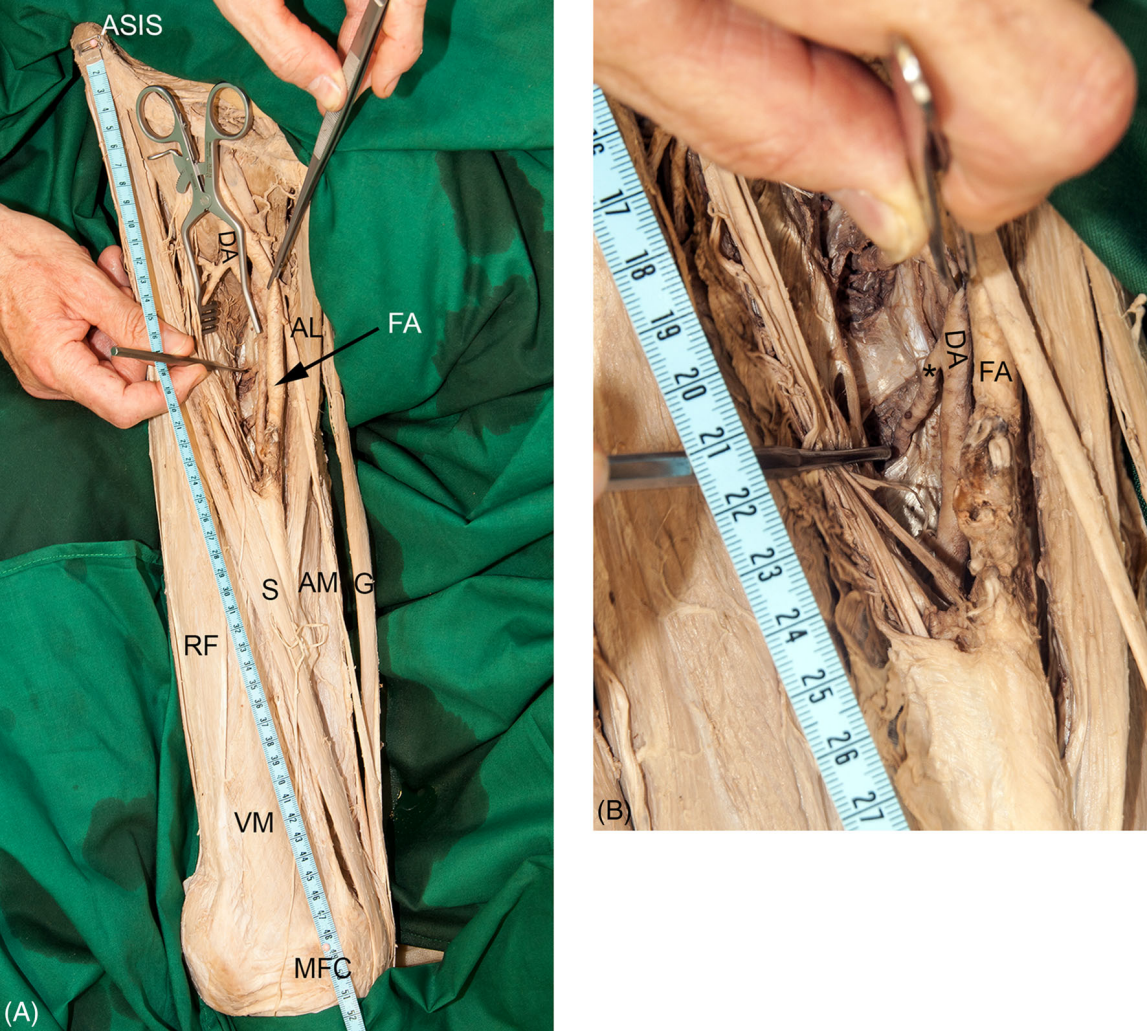
**A‐B** The photographs exemplify the measurement of the passage of one perforating artery (asterisk). (**A**) The image shows the reference line between the anterior superior iliac spine (ASIS) and the medial femoral condyle (MFC). (**B**) The photograph gives a detailed view of the measurement using a probe. AL, adductor longus muscle; AM, adductor magnus muscle; DA, deep artery of thigh; FA, femoral artery; G, gracilis muscle; RF, rectus femoris muscle; S, sartorius muscle; VM, vastus medialis muscle. [Color figure can be viewed at http://wileyonlinelibrary.com]

We measured the leg length from the anterior superior iliac spine to the plantar aspect of the calcaneal tuberosity (Knussmann, 1988) using a commercial measuring tape. The levels of origin and passage near the body of the femur of all perforating arteries were measured along a line from the most ventral aspect of the anterior superior iliac spine to the most prominent part of the medial femoral condyle. We fixed the measuring tape with pins on these two bony landmarks and used a transversally held probe to determine the level of origin and passage with an accuracy of 0.5 cm (Fig. 2). This seems to be adequate for determining danger zones as well as for the reproducibility during clinical routine. All measurements were performed by the same person to avoid any interobservational errors.

### Statistics

Statistical analysis was performed with IBM SPSS Statistics 25 and R 3.5.1. For visualizing the danger zones along the body of the femur, we used histograms to show the frequency of arterial branches at different positions and provided line‐drawings using a photograph taken from one of the authors' legs. On the line‐drawings, we marked each arterial branch measured at the site of its passage close to the body of the femur in relation to the leg length, separated for sex and side. Furthermore, we evaluated systematic differences between male and female specimens as well as between left and right legs in the number of perforating arteries, the number of branches passing near the body of the femur, and in the difference between arteries originating and branches passing with mixed model Poisson regression. To adjust for correlation between the left and right legs of the same individual, a random intercept for each specimen was included.

## RESULTS

### Number of Perforating Arteries and Branches

**Figure 3:**
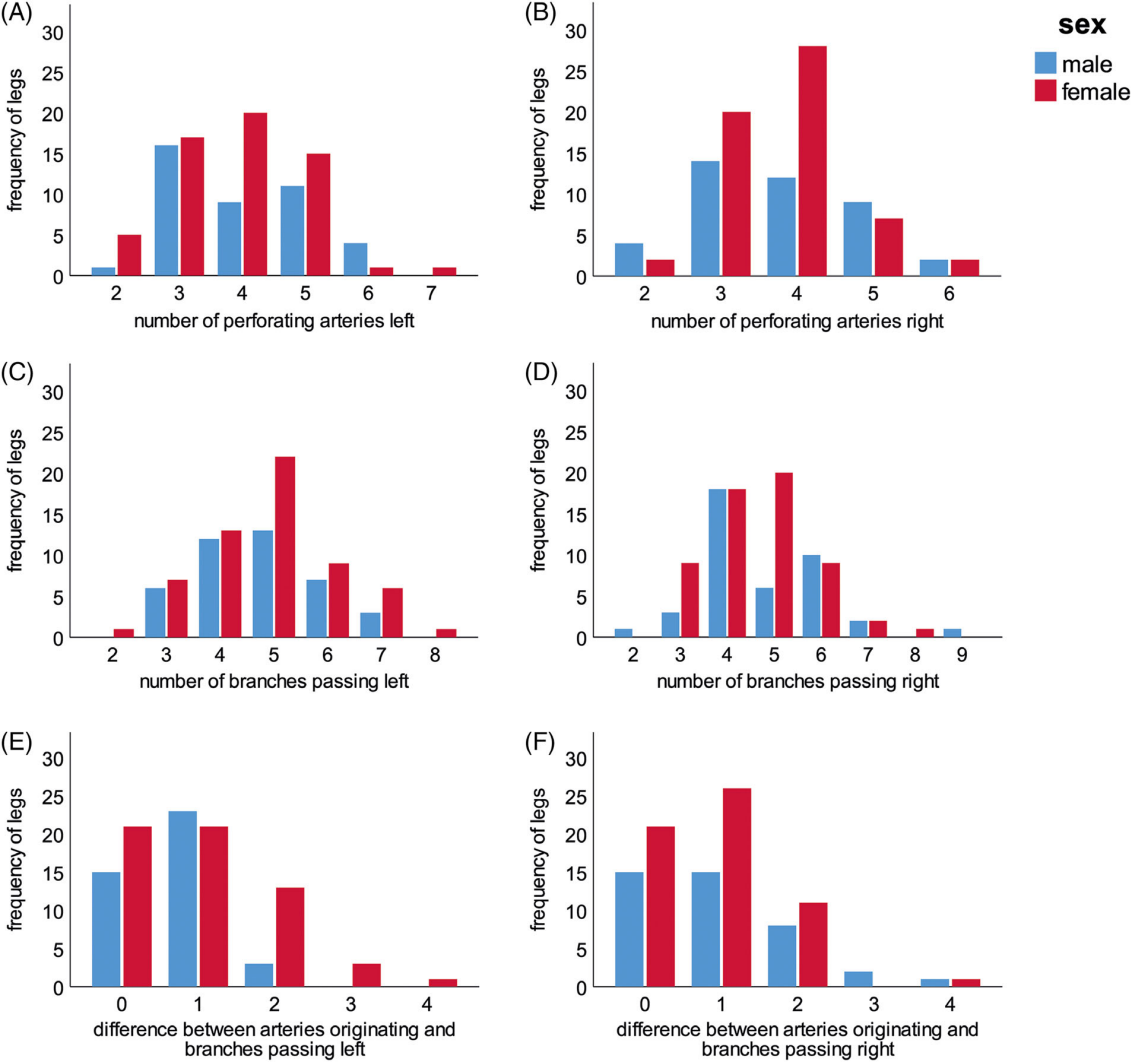
(**A–F**) The graphs show the frequencies of the different numbers of perforating arteries originating from the deep artery of thigh (**A,B**) of their branches subsequently passing between the tendons of the adductor muscles and the body of the femur (**C,D**) and of the intraindividual differences between the numbers of arteries originating and branches passing to the dorsal aspect of the thigh (**E,F**). The mixed model Poisson regression revealed no significant correlation in terms of sex and side. *n* = 100 (41 males, 59 females). [Color figure can be viewed at http://wileyonlinelibrary.com]

The number of perforating arteries originating from the deep artery of thigh varied between two and seven in all analyzed legs. The median was 4 for both sides and sexes. In 69 (34.5%), that is, the majority of all investigated legs, four perforating arteries originated from the deep artery of thigh, closely followed by 67 legs (33.5%) that contained three perforating arteries. In 42 (21%) cases, that is, in more than one‐fifth of all legs, five perforating arteries were found to be present, and in 12 legs (6%), merely two perforating arteries were found. In the remaining 10 cases (5%), either six or even seven arteries were present. Figure 3A,B shows the frequencies for the different number of perforating arteries for left and right legs considering the sex of the individuals. Neither sex nor side was significantly correlated with the number of perforating arteries (sex: *P* = 0.822, side: *P* = 0.541).

In 187 legs (93.5%), the terminal branch of the deep artery of thigh passed to the dorsal aspect close to the body of the femur, and was thus counted as a perforating artery. In the remaining cases, it terminated in or passed through the adductor muscles lacking any direct contact with the body of the femur. This occurred unilaterally in four female and three male specimens (five left and two right legs) and bilaterally in three females.

More often than not (128 legs, 64%), at least one perforating artery divided into two to four branches before entering the back of the thigh. The total number of those branches passing near the body of the femur varied between two and nine. The median was 5 for both sides in women and the left side in men and 4 for the right male legs. Most frequently, either four (61 legs, 30.5%) or five (61 legs, 30.5%) branches were present. In 35 legs (17.5%), six branches were observed, while in 25 legs (12.5%), there were only three of them passing through to the dorsal aspect of the thigh. In the remaining five cases (2.5%), two, eight, or even nine branches were observed. In Figure 3C,D, the frequency distribution of the numbers of branches is shown per sex and side. Again, neither sex nor side was significantly correlated with the number of arteries passing near the body of the femur (sex: *P* = 0.785, side: *P* = 0.698).

Only in 72 (36%) of all examined legs, the number of branches passing near the body of the femur was equal to that of perforating arteries originating from the deep artery of thigh. In 85 cases (42.5%), one more, and in 35 (17.5%), two more arteries passed near the body of the femur than originated from the deep artery of thigh. In the remaining eight cases (4%), the difference between the originating and passing arteries was three or four. In Figure 3E,F, the frequencies of the intraindividual differences between the number of perforating arteries and their branches is shown for males and females in both legs. There was no significant correlation in terms of sex (*P* = 0.276) and side (0.710).

### Safe Zones vs. Danger Zones

**Figure 4:**
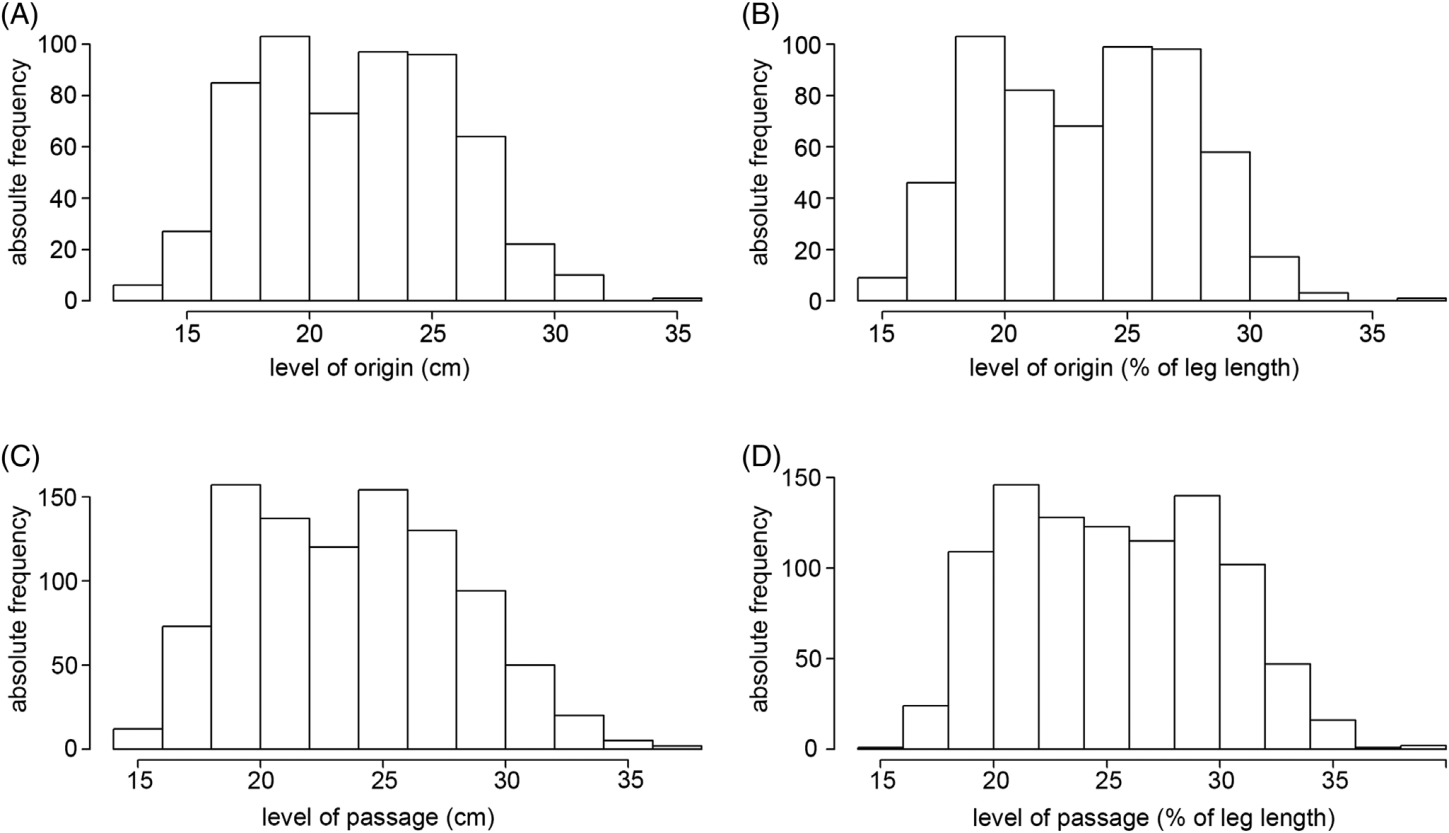
(**A–D**) No constant levels of origin can be given, as perforating arteries originated at every level between 12.5 and 34.5 cm from the anterior superior iliac spine, that is, 14–38% of the leg length (**A,B**). Between 14.0 and 36.5 cm from the anterior superior iliac spine (16–39% of the leg length), no safe zones can be predicted in which arteries most unlikely pass between the adductor tendons and the body of the femur (**C,D**). In each bar, the absolute number of all perforating arteries observed within a predefined range is given. In (**A**) and (**C**), each range represents 2 cm, whereas in (**B**) and (**D**), it is equal to 2% of leg length.

**Figure 5:**
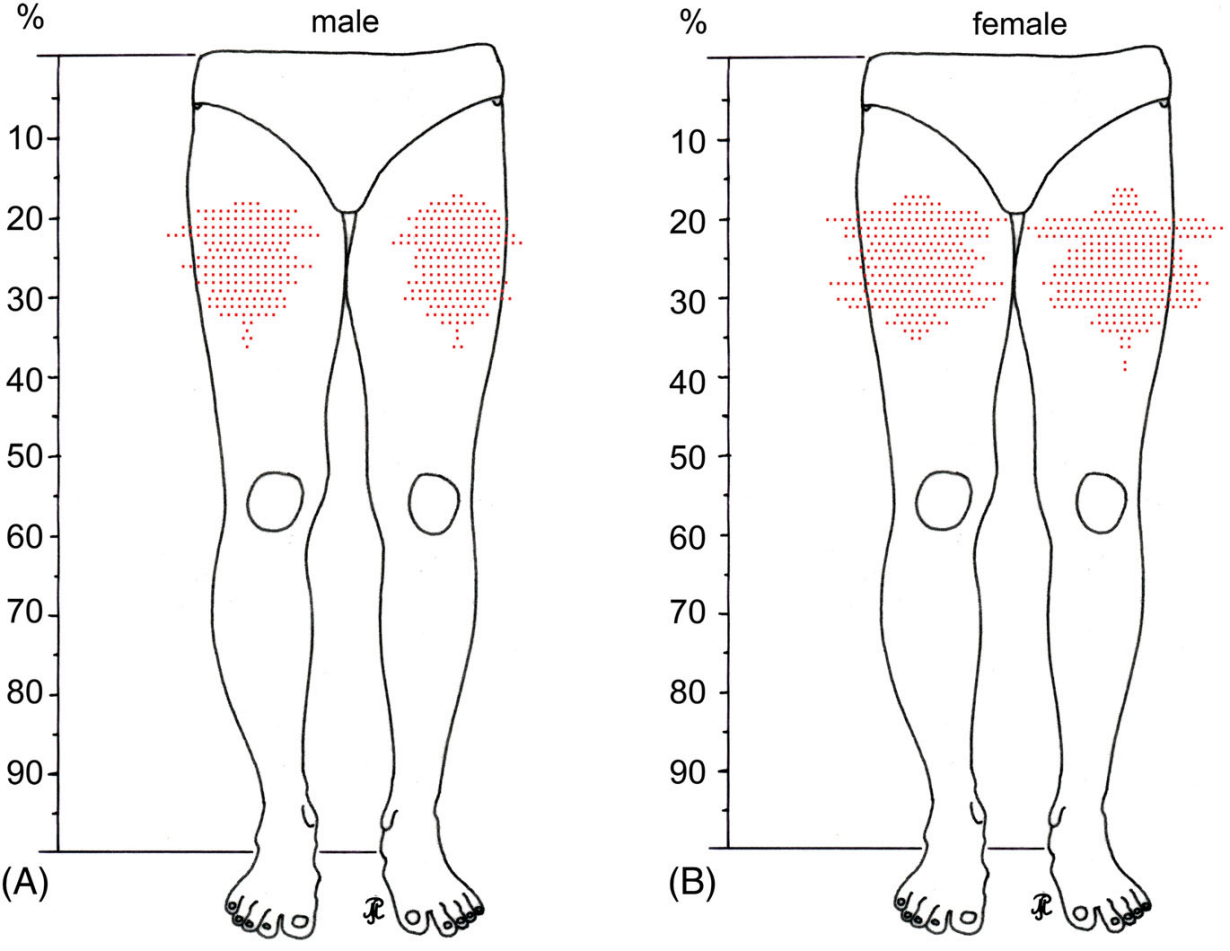
(**A,B**) The line drawings show the distribution pattern of perforating arteries passing between the adductor muscles and the body of the femur in percentage of the leg length. Each point represents a singular vessel. The figures summarize the findings in all male (*n* = 41) (**A**) and all female individuals (*n* = 59) (**B**) separated for left and right legs. [Color figure can be viewed at http://wileyonlinelibrary.com]

As in both sexes perforating arteries originated at every level between 12.5 and 34.5 cm from the anterior superior iliac spine (14–38% of leg length), no constant levels of origin can be given, neither in absolute distances nor in relation to the leg length (Fig. 4A,B). Subsequently, the arteries may follow an either horizontal or descending course. The perforating arteries or their branches passed to the back of the thigh at every level between 14.0 and 36.5 cm from the anterior superior iliac spine (16–39% of leg length; Fig. 4C,D). Within this distance, no safe zones along the body of the femur could be identified, neither in absolute numbers nor in relation to the leg length. Figure 5 shows all observed arteries on their level of passage near the body of the femur in relation to the leg length. It illustrates that a continuous danger zone exists between 16 and 39% of the leg length from the anterior superior iliac spine on both sides in males as well as in females.

### Additional Observations

**Figure 6:**
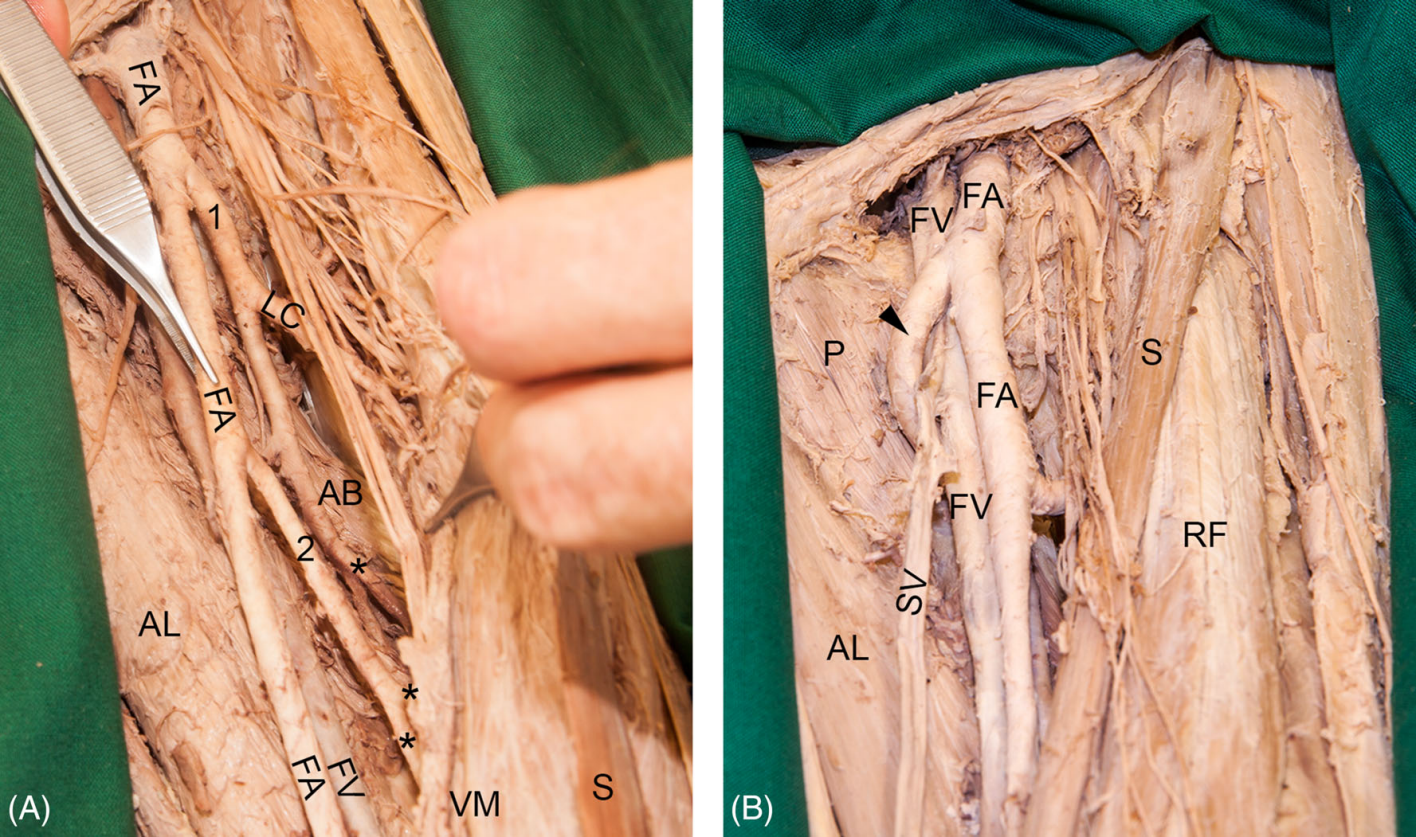
(**A**) The picture shows an example with two deep arteries of thigh instead of one in the left leg of a female individual. The regular deep artery of thigh (1) gave rise to the first perforating artery (asterisk). From the aberrant deep artery of thigh (2), the remaining perforating arteries (asterisks) arose. (**B**) In the left leg of a female individual, the deep artery of thigh (arrowhead) looped superficially around the femoral vein (FV) and great saphenous vein (SV). This situation may lead to problems during puncture or endovascular procedures of the femoral artery (FA) or FV. AB, adductor brevis muscle; AL, adductor longus muscle; P, pectineus muscle; RF, rectus femoris muscle, VM, vastus medialis muscle; S, sartorius muscle. [Color figure can be viewed at http://wileyonlinelibrary.com]

In three left legs (two males and one female), the femoral artery gave rise to two instead of one deep artery of thigh. In these cases, the first or the first and second perforating arteries were branches from the regular deep artery of thigh. The remaining perforating arteries originated from the aberrant second deep artery of thigh (Fig. 6A). In the left leg of one female, the deep artery of thigh coursed in an untypical manner: it originated from the medial side of the femoral artery and looped around the ventral aspect of the femoral and great saphenous vein before it coursed through the ground of the femoral triangle (Fig. 6B).

## DISCUSSION

Former data concerning the number of perforating arteries are nonconclusive and differ substantially from our results. Three to four perforating arteries, a number that is described as the regular situation in most textbooks of anatomy and surgery (Lang and Wachsmuth, 1972; Williams and Warwick, 1980; Netter, 1987; Hoppenfeld et al., 2009), have only been observed in 68% of the 200 legs examined in this study. Lipshutz (1916) and Adachi (1928) observed this arrangement in 79%. Farouk et al. (1999) reported a constant number of four perforating arteries in all of the 40 legs they investigated. Our data show that their number can vary between two and seven, which was also discussed by Dissanayake et al. (2016). On the other hand, Lipshutz (1916) reported the possibility of two to five and Adachi (1928) of only two to four perforating arteries. In our study, five perforating arteries have been observed in 21% of legs, which differs from the data of Lipshutz (1916), who has found five arteries in only 9% of cases.

As of yet, it had not been reported that the perforating arteries may branch before their passage through the tendinous arches of the adductor muscles. Only Adachi (1928) mentioned that their exact counting is ambiguous because of their frequent division at the site of their passage through the tendons of the adductor muscles. Here, for the first time, we report that in 64% of the observed legs, the number of arteries passing near the body of the femur exceeded those originating from the deep artery of thigh. Up to nine arterial branches can pass between the tendons of insertion of the adductor muscles and the body of the femur. This is an important observation, as the danger to the arteries due to surgical procedures is greater at that position than at their origin from the deep artery of thigh.

Former authors used different landmarks to localize perforating arteries. In living persons, it may be difficult to determine accurately the midpoint of the inguinal ligament (Siddharth et al., 1985) or the tip of the greater trochanter (Farouk et al., 1999), especially during surgical interventions. Two imaginary lines (Dissanayake et al., 2016) connecting bony landmarks that are partly difficult to palpate also do not seem to be particularly useful during clinical practice. Therefore, we used a line between two landmarks that are always palpable, even if the leg is covered by a blanket in the operating theater.

Although some authors tried to fix the origin of the perforating arteries (Siddharth et al., 1985; Dissanayake et al., 2016), or their passage near the body of the femur (Farouk et al., 1999), the present investigation showed that the position of the individual perforating arteries is quite variable. It was not possible to identify hotspots of these arteries, neither for their origin nor for their passage. Calculating mean values of the level of origin or passage of the individual arteries to describe their real position as done by former authors (Siddharth et al., 1985; Farouk et al., 1999; Dissanayake et al., 2016) has turned out impossible for our results. The source and course of the individual arteries are also dependent on the total number of perforating arteries and their respective number of branches in each individual leg of the same subject. For instance, the second perforating artery in one leg can be at the similar level as the third or fourth artery number in another leg. Therefore, we aimed to locate areas in which they likely pass near the body of the femur, realizing that there is one big danger zone between 14.0 and 36.5 cm measured from the anterior superior iliac spine. Siddharth et al. (1985) observed up to six perforating arteries, but calculated mean values for only four of them. Farouk et al. (1999) reported that the perforating arteries showed constant positions in relation to six parts of the length of the body of the femur. They observed all perforating arteries within the second to fourth, which is by no means accurate enough to locate the exact position during surgical procedures. Regarding their calculated mean values and ranges for the level of perforating arteries passing near the body of the femur, they also showed a high variability, despite their constant observation of four perforating arteries. As the range of the calculated mean values overlapped in both studies (Siddharth et al., 1985; Farouk et al., 1999), again such calculations seem to be of limited value. Perforating arteries and their branches could pass anywhere along the body of the femur between 16 and 39% of the leg length. Most likely, the reason seems to be that their number is variable and that in 64% of legs at least one of them subsequently divided in up to four branches passing close to the body of the femur. Furthermore, it is important to consider the terminal branch of the deep artery of thigh. In 93.5% of the legs examined in this study, it also reached the dorsal aspect of the thigh, frequently after branching into two or three arteries. In addition, the orientation of the perforating arteries toward the back of the thigh is variable; they may course in an almost horizontal or more descending manner.

This high variability of the perforating arteries could be explained by their embryogenesis. The deep artery of thigh is said to be derived from an arterial plexus around the femur in an embryo of a length of 14 mm (Senior, 1919) (Carnegie stage 17–18; week 6 [O'Rahilly and Müller, 1996]). In a 22‐mm‐long embryo (Carnegie stage 20–21, week 7 [O'Rahilly and Müller, 1996]), this artery gives origin to only one perforating artery that seems to represent the first or second of the adult series (Senior, 1919). This perforating artery is involved in an extensive arterial plexus, which is also connected with the remainder of the distal part of the embryonic sciatic artery located on the dorsal aspect of the thigh (Senior, 1919). Obviously, the other perforating arteries develop from this plexus in different manner. Therefore, they vary in number, origin, course, and branching pattern.

In all studies, including this one, only the arteries have been examined. As arteries are commonly accompanied by two veins, the likelihood to injure any blood vessel during surgical interventions near or on the body of the femur is even greater. However, harming a vein would not interrupt the blood supply of the body of the femur but may nevertheless lead postoperatively to an occult loss of blood.

In addition, we observed in one of our specimens the obviously rare but not unique case of a superficial loop of the deep artery of thigh, which has also been reported as early as the 19th century (Friedlowsky, 1867). Such a situation is a potential danger during puncture or endovascular procedures of the femoral vein or femoral artery and underscores the relevance of being aware of the variability of all the arteries of the thigh. As this topic is beyond the aim of our study, the reader is referred to former publications (Lipshutz, 1916; Siddharth et al., 1985; Massoud and Fletcher, 1997).

Our results underline the fact that surgeons have to keep in mind that perforating arteries may occur within the operation field and that their exact position cannot be predicted. Thus, these arteries may be prone to injury. Especially during plate osteosynthesis or cerclage wiring, the formation of pseudoaneurysms, major lesions of the vessel wall, or vascular occlusions have been reported. This may even lead to severe consequences for the patient (Neubauer et al., 2016; Devendra et al., 2018). Apivatthakakul (2013) showed that perforating arteries were interrupted in almost one quarter of percutaneous cerclage wiring simulated on anatomic specimens. Vascular injuries are also known complications during surgical fixation of the pins of an Ilizarov apparatus or of a necessary osteotomy of the femur. A postoperative formation of a pseudoaneurysm can be due to the proximity of such a pin to an artery (Paley, 1989; Goldstein et al., 2013). From the anatomical point of view, approaches without careful exposure of the highly variable perforating arteries near the body of the femur seem to implicate a higher possibility of vascular complications.

## CONCLUSION

The present study was based on the dissection and measuring of perforating arteries in 200 legs and demonstrated the high variability of these arteries regarding number, point of origin, as well as level of passing in close proximity to the body of the femur. Thus, no specific safe zone could be identified, neither in absolute terms nor in relation to the leg length. Within 14.0 and 36.5 cm (16–39% of the leg length) from the anterior superior iliac spine, up to nine perforating arteries, respectively, their branches may course posteromedially close to the body of the femur.
